# Growth-related trophic changes of *Thunnus thynnus* as evidenced by stable nitrogen isotopic values in the first dorsal spine

**DOI:** 10.1038/s41598-020-66566-w

**Published:** 2020-06-18

**Authors:** Paola Rumolo, Angelo Bonanno, Simona Genovese, Teresa Romeo, Salvatore Mazzola, Gualtiero Basilone, Serena Gherardi, Pietro Battaglia, Franco Andaloro, Marco Barra

**Affiliations:** 1 CNR-ISMAR, Calata Porta di Massa (Interno Porto di Napoli), Naples, Italy; 2 CNR-IAS, Torretta Granitola, Campobello di Mazara (TP), Italy; 3 Stazione Zoologica Anton Dohrn, Centro Interdipartimentale della Sicilia, Milazzo (ME), Italy; 4 Istituto Superiore per la Protezione e la Ricerca Ambientale, ISPRA, Milazzo (ME), Italy

**Keywords:** Biochemistry, Ecology

## Abstract

The bluefin tuna*, Thunnus thynnus*, is a highly migratory and long-living fish at the top of the pelagic food web. As top predator, it plays a key role in the stability of marine food webs by exerting top-down control on its prey. The diet composition of bluefin tuna varies in relation to its growth, seasons and migratory patterns, making it difficult to evaluate spatial and temporal effects. This latter aspect is further complicated to be determined during the first months of life, when *T. thynnus* specimens have a rapid growth rate leading to changes in the trophic status. In this study, the potential collagen-related effects on δ^15^N and δ^13^C values were evaluated on the whole spine of adult tuna specimens collected in the central Mediterranean Sea. Obtained results showed non-significant differences between extracted and non-extracted collagen samples for δ^15^N in whole spine, allowing adopting the isotopic analysis both for *annuli* in the spine section of adults and for younger specimens, whose spine size does not permit the collagen extraction. Specifically, isotopic analysis of whole spine of the young of the year specimens, showed a rapid change in δ^15^N values with length, following an exponential model. For older specimens, δ^15^N values were higher and varied around a plateau, likely due to a higher specificity in the choice of prey and/or to change in the geographical location. Such variability was also mirrored in *annuli* of spines sections of adult tunas. As far as δ^13^C values are concerned, a strong collagen-related effect was evidenced, likely highlighting the influence of lipids. Consequently, δ^13^C analysis may be used only on adult specimens where collagen extraction is possible. This research also showed how isotopic analysis of both whole sample and sequence of *annuli* in the cross-section of dorsal spine might produce isotopic profiles useful to detect specific trophic dynamics along the bluefin tuna growth.

## Introduction

In recent years, stable nitrogen and carbon isotopes analyses (δ^15^N and δ^13^C), performed on less metabolically active tissues, such as scale, otoliths, spines and bones, allowed to investigate several aspects of animal’s feeding habits, habitat use and migration (e.g. ^1–6^). However, there are different isotopic approaches for estimating diet and movement patterns of multiple taxa using inert tissues. For instance, the collagen extracted from whole bone of an animal can provide an integration of stable isotopes from multiple years with a general interpretation of its trophic behavior during the entire lifetime^2,7–11^. The analysis in single layers in some hard structures, such as teeth and humerus (with each layer representing a different interval of animal’s life), provides a time series of isotopic data, useful for reconstructing an animal’s foraging ecology over time^7,12–20^. It is important to single out that some methods need large amount of sample in order to remove lipids and other contaminants (*e.g*. collagen extraction), thus preventing their use for samples characterized by small dimensions^14^; other methods require a greater efficiency and accuracy in the determination and in the selection of individual annual growth layer from the tissue section (*e.g*. skeletochronology method)^14–16^.

The present study proposes a method able to estimate stable isotope values in metabolically non-active tissues (with a life history memory), verifying if it could be used also when the amount of sample material is not enough to perform the traditional collagen extraction procedure, (such as in very small fish species, in archaeological remains or in hard tissues of species stored in museums).

The isotopic analysis was here applied on the first dorsal spine of both adults and young of the year (YOY) bluefin tuna specimens, caught in the central Mediterranean Sea, to address important questions on the ecology and feeding behaviour of this species during its growth. Spine sections of adult tuna, often chosen for growth studies (e.g.^20^), were also considered to combine isotopic results with growth layers.

The bluefin tuna*, Thunnus thynnus*, is a highly migratory and long-living fish at the top of the pelagic food web and plays a key role in the stability of marine food webs by exerting top-down control on its prey^21^. It feeds on a broad spectrum of prey items, such as small pelagic fish, cephalopods and shrimps^22–25^; furthermore, in some Mediterranean and Atlantic feeding grounds, the component of mesopelagic fish is dominant in its diet^26,27^. However, the diet composition of bluefin tuna varies in relation to its growth, seasons and migratory patterns, making it difficult to evaluate spatial and temporal effects. This latter aspect is further complicated during the first months of life, when *T. thynnus* specimens have a rapid growth rate leading to changes in its trophic status. Moreover, the Reg. CE 302/2009 prohibits fishing tuna specimens of size smaller than 115 cm (specimen weight <30 kg), making the trophic position in the early life stage difficult to be determined.

In the present study, stable nitrogen and carbon isotopes (δ^15^N and δ^13^C) values and elemental nitrogen (%N) and carbon (%C) composition were obtained from: (1) extracted and non-extracted collagen samples obtained from the same spines of bluefin tuna adults, in order to evaluate the possible differences in the isotopic values between the two approaches; (2) whole spine of both YOY and adults tuna (without collagen extraction), to highlight differences in feeding behavior between the two macro-size classes (YOY and Juveniles/Adults); (3) untreated micromilled powder obtained from individual growth layers of spine section, to provide a more detailed trophic ecology in each layer representing a different period of animal’s stage.

## Results

**Figure 1:**
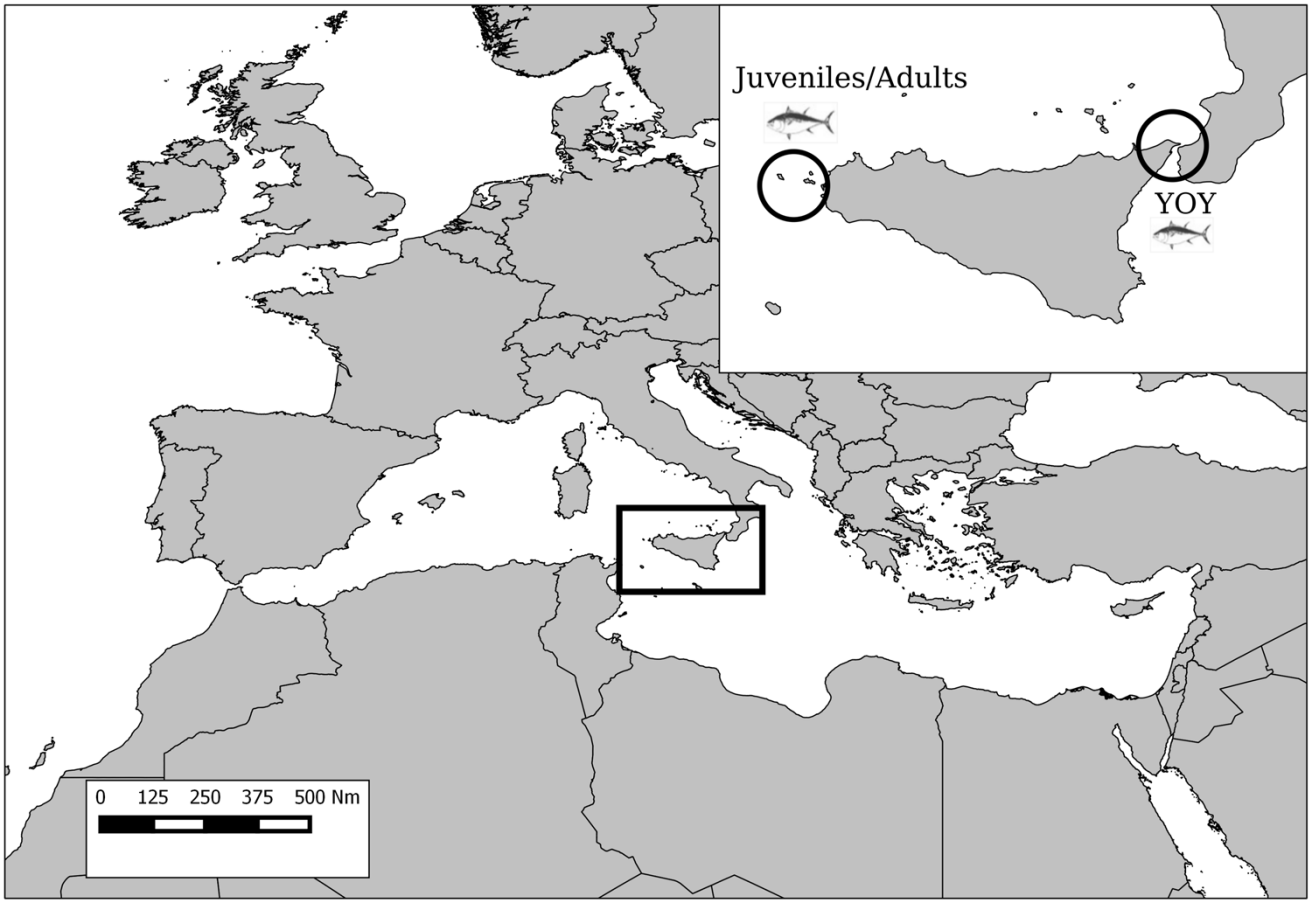
Study area with sampling locations (Map generated by using the QGis 2.8 - https://qgis.org/).

A total of 51 *T. thynnus* specimens were collected in two sampling sites (Fig. 1): 29 Juveniles/Adults (with fork length FL > 50 cm) and 22 young of the year (YOY with FL < 50 cm) tuna. Ages of the specimens ranged between the first year of life to fifteen years (Table 1).

**Table 1:** δ^13^C and δ^15^N values for extracted and non-extracted collagen samples. The “C” label refers to the results of the isotopic analysis on the extracted collagen samples.

ID	Label	Area	LF (cm)	Weight (Kg)	Estimated age	δ^15^N vs AIR	sd	δ^13^C vs PDB	sd
YOY1		North-eastern of Sicily	17.7	0.1	<1 year	2.9	0.0	−14.9	0.4
YOY2		North-eastern of Sicily	19.0	0.1	<1 year	4.3	0.1	−16.3	0.1
YOY3		North-eastern of Sicily	25.2	0.3	<1 year	4.7	0.2	−15.3	0.1
YOY4		North-eastern of Sicily	25.6	0.3	<1 year	5.2	0.1	−16.6	0.3
YOY5		North-eastern of Sicily	27.8	3.9	<1 year	4.9	0.2	−18.0	0.4
YOY6		North-eastern of Sicily	30.5	4.6	<1 year	5.8	—	−17.4	—
YOY7		North-eastern of Sicily	31.2	5.2	<1 year	5.5	—	−19.2	—
YOY8		North-eastern of Sicily	31.7	5.7	<1 year	6.1	1.1	−21.6	0.5
YOY9		North-eastern of Sicily	32.9	6.4	<1 year	4.8	1.0	−19.4	0.5
YOY10		North-eastern of Sicily	36.6	9.5	<1 year	6.2	0.1	−21.2	0.1
YOY11		North-eastern of Sicily	37.4	10.3	<1 year	5.0	0.6	−21.0	1.4
YOY12		North-eastern of Sicily	38.0	11.1	<1 year	6.0	—	−20.0	—
YOY13		North-eastern of Sicily	38.9	11.6	<1 year	6.3	0.1	−19.1	1.5
YOY14		North-eastern of Sicily	42.8	15.5	<1 year	5.9	0.5	−21.1	1.4
YOY15		North-eastern of Sicily	43.0	16.5	<1 year	6.3	—	−21.6	—
YOY16		North-eastern of Sicily	44.0	26.6	<1 year	5.0	—	−21.8	—
YOY17		North-eastern of Sicily	44.2	19.5	<1 year	6.1	2.1	−20.8	1.9
YOY18		North-eastern of Sicily	46.5	28.4	<1 year	4.9	0.3	−19.6	0.5
YOY19		North-eastern of Sicily	47.4	26.5	<1 year	6.1	2.4	−20.8	1.2
YOY20		North-eastern of Sicily	47.4	2.2	<1 year	6.6	0.0	−19.3	0.1
YOY21		North-eastern of Sicily	48.0	2.2	<1 year	6.7	0.1	−17.7	0.3
YOY22		North-eastern of Sicily	48.0	32.9	<1 year	5.9	0.1	−20.7	0.3
Juvenile/Adult 1		North-western of Sicily	51.4	35.3	N.A.	6.3	0.7	−19.6	0.2
Juvenile/Adult 2		North-western of Sicily	119.0	24.0	4 year	7.2	0.4	−19.1	—
Juvenile/Adult 3		North-western of Sicily	121.0	28.0	N.A.	7.7	—	−18.0	—
Juvenile/Adult 4		North-western of Sicily	123.0	29.0	N.A.	5.3	0.7	−12.8	1.1
Juvenile/Adult 5		North-western of Sicily	124.0	30.0	4 year	6.3	0.2	−15.8	0.4
Juvenile/Adult 6		North-western of Sicily	132.0	31.0	N.A.	7.2	0.5	−15.9	1.1
Juvenile/Adult 7		North-western of Sicily	132.0	35.0	7 year	6.5	0.3	−21.9	3.8
Juvenile/Adult 8		North-western of Sicily	135.0	34.0	7 year	5.8	0.9	−20.2	0.4
Juvenile/Adult 9		North-western of Sicily	137.0	35.0	7 year	7.0	0.9	−20.1	0.9
Juvenile/Adult 10		North-western of Sicily	137.0	33.0	7 year	12.2	0.2	−16.0	1.7
Juvenile/Adult 11		North-western of Sicily	138.0	34.0	7 year	7.1	0.1	−15.0	1.5
Juvenile/Adult 12		North-western of Sicily	139.0	42.0	6 year	7.0	0.3	−19.5	1.0
Juvenile/Adult 13		North-western of Sicily	142.0	37.0	6 year	6.1	0.4	−16.5	0.3
Juvenile/Adult 14		North-western of Sicily	143.0	39.0	6 year	9.1	0.3	−19.8	—
Juvenile/Adult 15		North-western of Sicily	144.0	39.0	N.A.	7.1	0.6	−23.4	1.4
Juvenile/Adult 16		North-western of Sicily	147.0	45.0	6–7 year	7.6	0.3	−16.9	—
Juvenile/Adult 17		North-western of Sicily	148.0	40.0	N.A.	7.7	1.2	−19.3	1.6
Juvenile/Adult 18		North-western of Sicily	152.0	47.0	N.A.	5.8	0.6	−21.0	0.9
Juvenile/Adult 19		North-western of Sicily	152.0	58.0	N.A.	10.4	0.5	−18.8	1.8
Juvenile/Adult 20		North-western of Sicily	153.0	47.0	6–7 year	8.1	0.5	−18.4	0.3
Juvenile/Adult 21		North-western of Sicily	153.0	47.0	6–7 year	6.1	0.5	−16.4	2.1
Juvenile/Adult 22		North-western of Sicily	155.0	48.0	7 year	8.1	1.6	−15.3	—
Juvenile/Adult 23		North-western of Sicily	117.0	27.0	5 year	8.2	0.2	−15.2	0.3
Juvenile/Adult 24		North-western of Sicily	136.0	76.0	7 year	7.8	0.2	−14.0	0.6
Juvenile/Adult 25		North-western of Sicily	162.0	74.0	6 year	7.7	0.2	−13.0	0.2
Juvenile/Adult 26		North-western of Sicily	172.0	141.0	10 year	9.3	0.1	−18.3	0.2
Juvenile/Adult 27		North-western of Sicily	178.0	87.0	9 year	7.6	0.1	−16.5	0.2
Juvenile/Adult 28		North-western of Sicily	202.0	138.0	10 year	6.8	0.2	−13.2	0.3
Juvenile/Adult 29		North-western of Sicily	212.0	170.0	15 year	8.2	0.1	−14.2	0.2
Juvenile/Adult 2C	C	North-western of Sicily	119.0	24.0	4 year	7.9	0.1	−14.2	0.1
Juvenile/Adult 3C	C	North-western of Sicily	121.0	28.0	N.A.	6.4	0.1	−12.7	0.0
Juvenile/Adult 4C	C	North-western of Sicily	123.0	29.0	N.A.	5.6	0.0	−11.5	0.1
Juvenile/Adult 5C	C	North-western of Sicily	124.0	30.0	4 year	6.3	0.1	−12.7	0.1
Juvenile/Adult 6C	C	North-western of Sicily	132.0	31.0	N.A.	5.4	0.1	−12.0	0.0
Juvenile/Adult 7C	C	North-western of Sicily	132.0	35.0	7 year	6.7	—	−15.6	—
Juvenile/Adult 8C	C	North-western of Sicily	135.0	34.0	7 year	5.9	0.0	−13.4	0.3
Juvenile/Adult 9C	C	North-western of Sicily	137.0	35.0	7 year	7.0	0.0	−14.1	0.1
Juvenile/Adult 10C	C	North-western of Sicily	137.0	33.0	7 year	10.6	0.0	−13.0	0.1
Juvenile/Adult 11C	C	North-western of Sicily	138.0	34.0	7 year	6.4	0.2	−12.6	0.0
Juvenile/Adult 12C	C	North-western of Sicily	139.0	42.0	6 year	6.2	0.0	−12.7	0.1
Juvenile/Adult 13C	C	North-western of Sicily	142.0	37.0	6 year	6.9	0.0	−12.3	0.0
Juvenile/Adult 14C	C	North-western of Sicily	143.0	39.0	6 year	7.9	0.0	−12.3	0.0
Juvenile/Adult 15C	C	North-western of Sicily	144.0	39.0	N.A.	7.3	0.1	−13.2	0.1
Juvenile/Adult 16C	C	North-western of Sicily	147.0	45.0	6–7 year	7.7	0.1	−13.6	0.0
Juvenile/Adult 17C	C	North-western of Sicily	148.0	40.0	N.A.	6.3	0.0	−12.7	0.0
Juvenile/Adult 18C	C	North-western of Sicily	152.0	47.0	N.A.	8.0	0.1	−12.8	0.1
Juvenile/Adult 19C	C	North-western of Sicily	152.0	58.0	N.A.	10.5	0.1	−13.3	0.0
Juvenile/Adult 20C	C	North-western of Sicily	153.0	47.0	6–7 year	7.1	0.0	−14.0	0.1
Juvenile/Adult 21C	C	North-western of Sicily	153.0	47.0	6–7 year	5.3	0.0	−12.4	0.0
Juvenile/Adult 22C	C	North-western of Sicily	155.0	48.0	7 year	6.6	0.2	−12.6	0.1
Juvenile/Adult 23C	C	North-western of Sicily	117.0	27.0	5 year	8.6	0.2	−17.6	0.7
Juvenile/Adult 24C	C	North-western of Sicily	136.0	76.0	7 year	7.8	0.2	−17.3	0.0
Juvenile/Adult 25C	C	North-western of Sicily	162.0	74.0	6 year	8.4	0.1	−18.4	0.5
Juvenile/Adult 26C	C	North-western of Sicily	172.0	141.0	10 year	9.9	0.1	−20.1	0.5
Juvenile/Adult 27C	C	North-western of Sicily	178.0	87.0	9 year	7.4	—	−17.8	—
Juvenile/Adult 28C	C	North-western of Sicily	202.0	138.0	10 year	7.8	0.5	−17.0	0.5
Juvenile/Adult 29C	C	North-western of Sicily	212.0	170.0	15 year	8.2	—	−15.1	—

**Figure 2:**
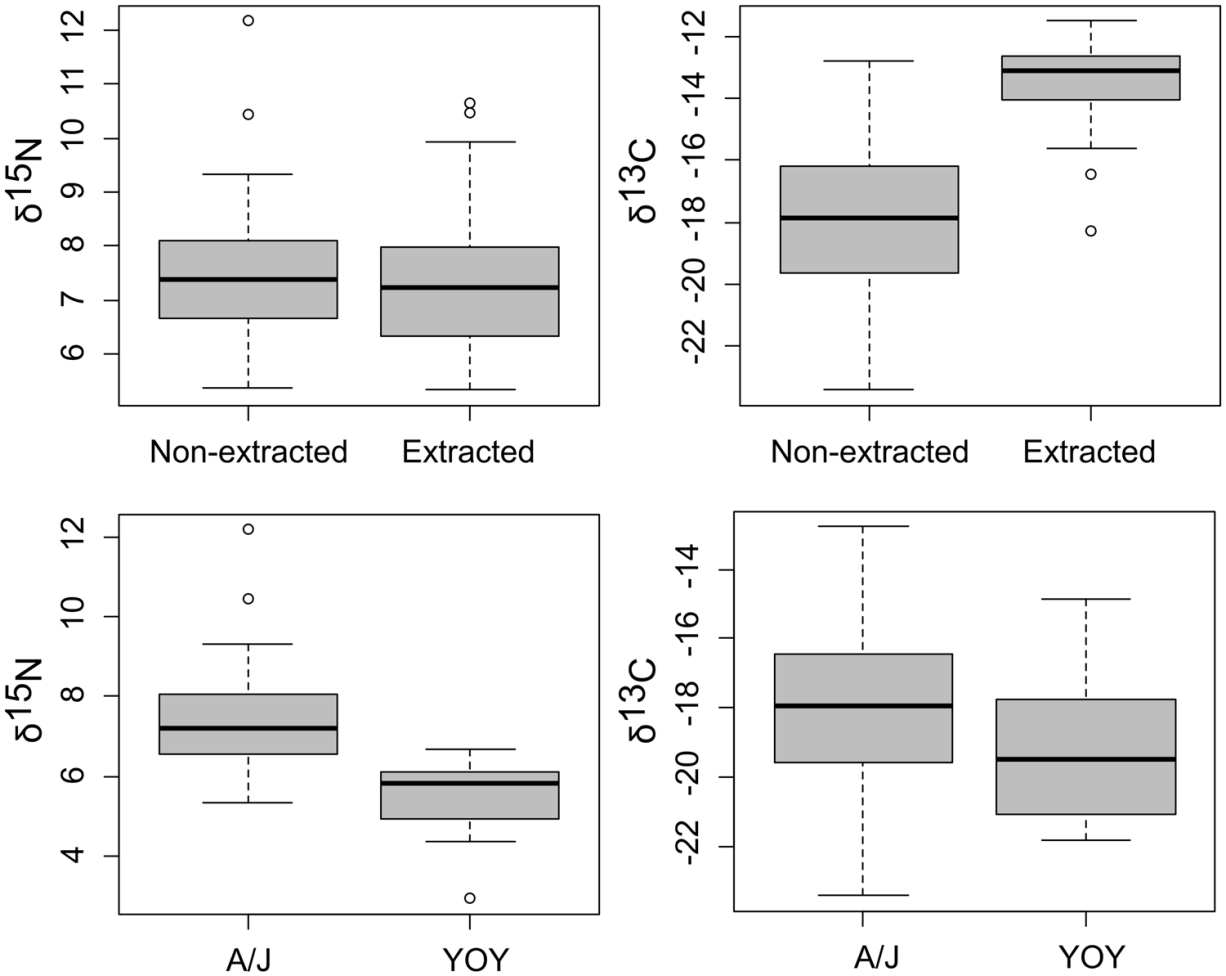
Boxplot of δ^13^C and δ^15^N values from spines of *Thunnus thynnus*. Top panels report the boxplots only for samples where it was possible to replicate the isotopic analyses on both extracted and non-extracted samples. Bottom panels boxplot compares the isotopic values between Adults/Juveniles (A/J) and young of the year (YOY) considering only non-extracted samples.

Paired Wilcoxon test, carried out on δ^15^N and δ^13^C values obtained on extracted and non-extracted collagen samples of 28 Juveniles/Adults tunas (Fig. 2), showed non-significant differences for δ^15^N values, while a significant difference was observed for δ^13^C (W = 0, p-value = 7.451e-09). Mean δ^13^C values of non-extracted collagen was 4.8‰ lower than the same extracted collagen samples (Fig. 2).

**Figure 3:**
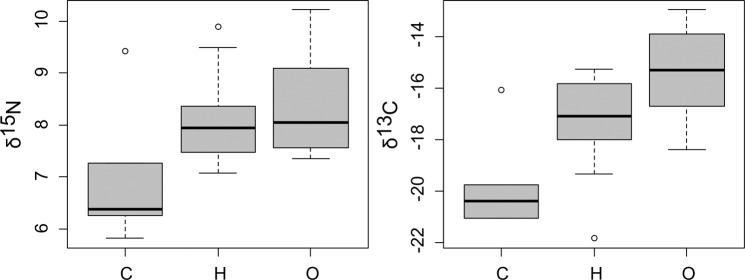
Boxplot of δ^13^C and δ^15^N values estimated in core (C), opaque (O) and non-opaque (H) bands in spine of *Thunnus thynnus* sections.

Looking at differences between YOY and Juveniles/Adults (only for non-extracted collagen samples), significant differences were found in terms of δ^15^N (W = 55, p-value = 5.384e-07), while non-significant difference was evidenced for δ^13^C values. In particular, δ^15^N values were higher in Juveniles/Adults specimens than in YOY, with absolute differences in median values of 1.4‰ (see Fig. 2). Finally, when δ^15^N and δ^13^C values were compared among core, non-opaque and opaque bands in spines section, non-significant differences were found in terms of δ^15^N, while significant differences were observed for δ^13^C. In particular, a Tukey HSD *post-hoc* test highlighted that δ^13^C values in the core were significantly lower (p < 0.05) than the ones recorded in non-opaque and opaque bands, while non-significant differences were identified between these latter categories (Fig. 3).

**Figure 4:**
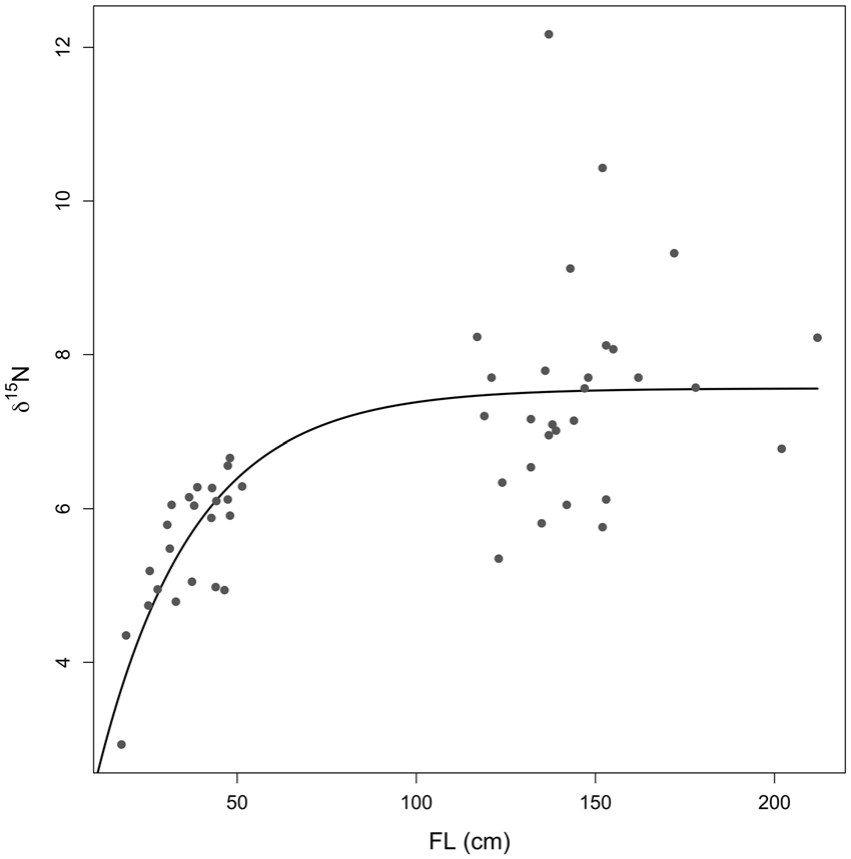
Exponential model fitted to δ^15^N values recorded in spines of *Thunnus thynnus* in relation to FL. According to the following equation, ${\delta }^{15}N=a\ast (1-{e}^{(-FL/b)}),$ the estimated parameters were: a = 7.566‰ and b = 26.75 cm.

In order to better characterize the change in δ^15^N values due to growth, δ^15^N values were fitted according to the exponential model (Fig. 4), the estimated parameters “a” and “b” were found to be 7.56‰ and 26.75 cm respectively (Table 2). In this context, the “a” parameter represents the δ^15^N plateau value, while the “b” one represents the 1/3 of the FL value where the plateau is reached. Thus, according to the fitted model, the plateau was reached at around 80 cm (FL).

**Table 2:** Exponential fitting estimates.

	Estimate	Std. Error	t value	Pr(>|t|)	*p*
a	7.5633	0.2277	33.222	<2e-16	0.001
b	26.7463	3.1649	8.451	3.94E-11	0.001

Residual standard error: 1.136 on 49 degrees of freedom.

Number of iterations to convergence: 5.

Achieved convergence tolerance: 7.88e-06.

## Discussion and conclusions

In isotopic studies focusing on hard tissues, a large amount of material is needed to carry out the collagen extraction in order to obtain reliable information on isotopes values^14^. In this study, we compared the results of isotopic analysis on dorsal spines of Bluefin tuna in order to support the adoption of isotopic analysis also when the amount of sample material is not enough to perform the traditional collagen extraction. Specifically, the non-significant difference between δ^15^N values obtained from extracted and non-extracted collagen samples permitted the direct determination of δ^15^N on whole spine of both YOY and Juveniles/Adults, and on milled bone powder (obtained from growth layer of a section spine).

δ^15^N values in consumer tissues are driven not only by trophic position of their prey, but are also strongly influenced by the baseline for δ^15^N (i.e. that of primary producers), which has a strong geographic variation^28,29^. In this study, we assumed that the YOY tuna, collected from north-eastern Sicily, were likely too young to have migrated in other sites^30^ and were assumed to have consumed their prey where they were caught. In this way, we did not consider the spatial effects on the δ^15^N values for smaller individuals. In this context, it is worth noting that YOY observations are quite close to the curve obtained by fitting the exponential model using δ^15^N and FL values (Fig. 4). On the contrary, adults’ δ^15^N values showed high dispersion around the plateau, evidencing a clear spatial effect (i.e. the observed higher variability could be likely due to changes in prey items reflecting higher migratory capability of adults, leading them to explore different areas in a relatively short time interval). Thus, assuming the lack of a spatial effect for YOY, the rapid increase of δ^15^N values in the size range 17–48 cm FL was likely linked to the trophic status of the consumed prey that changes quickly during the period of rapid growth for bluefin tuna (e.g. ^21,31,32^). In the first life stages (FL in the range 17–48 cm), the low δ^15^N values (mean 5.1 ± 1.4‰) recorded in the YOY tuna specimens may be due to a diet starting on planktonic copepods and euphausiids, that could provide a high-energy food source for juvenile bluefin tuna during periods of rapid growth, and subsequently on small cephalopods^22,30,31,33,34^. Zooplankton may represent an overlooked prey base for YOY bluefin tuna in the Mediterranean Sea but information on the diet of such early juveniles is scanty (e.g. ^22,30^). Moreover, at the best of our knowledge, this is the first study evaluating the relationship between δ^15^N values and Fork Length (FL) in the Young Of Year (YOY) bluefin tuna. Our data clearly evidenced a continuous increase of δ^15^N values with growth. The comparison between the dynamics of trophic shift in BFT and the one observed in *Thunnus albacares* evidenced strong differences mainly for smaller specimens. Weng *et al*. (2015) and Graham *et al*. (2007) reported a sigmoid relationship between δ^15^N values and FL, thus smaller yellowfin tuna (FL range 20–40 cm) showed an almost constant δ^15^N values up to FLs > 40 cm. For this species, the diet shift was very rapid and the δ^15^N plateau was reached between 40 and 50 cm. The δ^15^N values of bluefin tuna YOY (in this study) were characterized by a continuous increase up to 80 cm (FL). We believe that this difference between the two species could be linked to different factors such as: 1) the same prey in different waters may exhibit different stable isotopes values due to differences in the nutrient sources that affect the N values of food webs; 2) differences in physiology and development (e.g. differences in the development of gape size); 3) differences in feeding strategies and/or prey selectivity.

**Figure 5:**
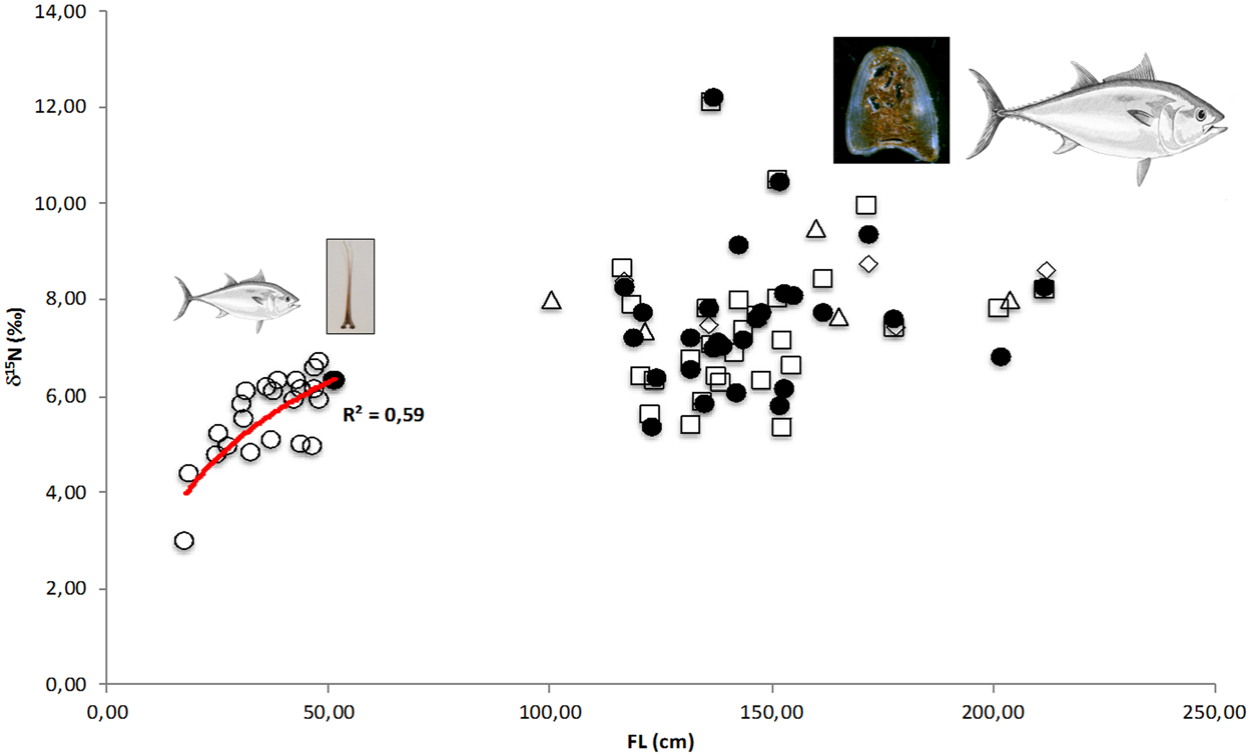
δ^15^N analysis of: Back-calculated size estimates from bands (FL at time t ◄ and at time t-1 Δ); *Whole* spine (YOY ○ and Juvenile/Adult ●) and 3. Extracted collagen □. (*Thunnus thynnus* picture permission granted by the author Andrea Paoli*)*.

On the other hand, the high variability found in δ^15^N values of Juveniles/Adults bluefin tuna suggested that for tuna with FL > 50 cm diet passes to be based mainly on cephalopods and fish^21,30^. Furthermore, this variability may be affected by geographical location of prey and by different prey assemblages. Indeed, during migration, from spawning locations to feeding grounds, bluefin tuna probably fed as they encountered patches of prey^26^ but it will be also possible that groups of migratory bluefin tuna stop to feed at unidentified locations during seasonal migrations (*e.g*. ^21^). The high variability of δ^15^N values in Juveniles/Adults specimens was also shown when the isotopic values, related to consecutive opaque and hyaline bands, were considered (Fig. 5).

When direct determination of δ^15^N was obtained on powder micromilled growth layers of spine of adults bluefin tuna, the amount of material did not permit to determine elemental composition of nitrogen (and consequently of C:N ratios), inhibiting also the evaluation of lipids effects in the growth layers.

The significant differences in δ^13^C values, observed by comparing isotopic values from extracted and non-extracted collagen from spines of Juveniles/Adults tuna, is due to the presence of lipids and bioapatite that can enrich or deplete these values, respectively. Despite sub-samples of spines were subject to acidification with HCl before the isotopic analysis and no effect was recorded during this process, we assumed that the effect of bioapatite on δ^13^C values could be negligible in our samples, in agreement to^16^. On the contrary, the δ^13^C values, lower in non-extracted collagen samples, evidenced the presence of lipid-bond carbon. Lipids are generally 2–3‰ more depleted in ^13^C than in other organic molecules^35,36^. Moreover, also when direct determination of δ^13^C values was obtained on powder micromilled growth layers of spine of adults bluefin tuna, Tukey HSD *post-hoc* test highlighted that δ^13^C values in the core were significantly lower (p < 0.05) than the ones recorded in the growth layers. Consequently, we recommend to consider only samples that fall in the C:N range 2.6–3.5^36^ to confirm that lipid extraction is not necessary. Since C:N ratio was not always determinable in untreated samples and, to our knowledge, no standard protocol for δ^13^C correction exists for *T. thynnus*, in this work we decided to use only the δ^15^N values to produce a time series of stable isotope patterns from individual animals. Indeed, both acidification and lipids removal should affect only bioapatite carbon and not collagen-bound nitrogen. Furthermore, the lipid molecules do not contain nitrogen^16^.

However, supplementary researches are needed to establish if the lipids effects are linked only to the inner medullary cavity or also to the different growth layers, especially for the case in which the C:N is difficult to determine due to samples material derived from drilled growth layers. Unfortunately, with our current knowledge, we are not able to evaluate this aspect. We postpone to future studies the possibility to find a method (or an equation) that will allow the correction of the lipids effects of δ^13^C values from untreated and not delipidated samples (whole spine and/or powder extracted from growth layers) for this species. However, the obtained results evidenced that the δ^15^N analysis can be used to produce isotopic profiles useful to detect trophic and foraging habitat changes along the individual lifetime, particularly when the amount of sample material is not enough to perform the collagen extraction, such as in small fish, archeological remains (mainly fish remains) and/or spines of fish stored in the museum (extinct species or in need of conservation).

## Materials and Methods

### Sample collection and processing

Bluefin tuna specimens were collected in two sampling sites located in the north-western and north-eastern Sicily (Italy), respectively (Fig. 1). No use of live animals was made in this study. Samples have been collected within a National Project funded by MIPAF (“Valuation of the ecotoxicology impacts from old and new contaminants, study of biology and ecology of the Mediterranean population of swordfish, bluefin tuna and albacore” cod. 6A108) and additional samples were obtained by Maritime Authority during the bluefin tuna landings in the designated port of Milazzo (ICCAT List Number 849). Sampling site and date, fork length (FL) and weight of the fish specimens were recorded (Table 1). Taxonomic identification of individuals was done examining the morphological and meristic features in accordance with key characters reported by^37,38^.

**Figure 6:**
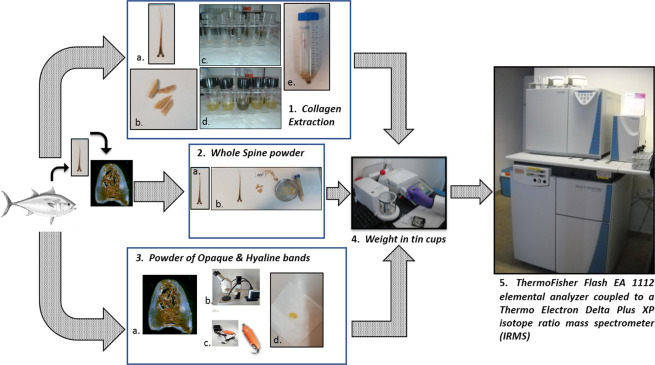
The work scheme of laboratory analysis. (1) Collagen extraction: (**a**) extraction of first dorsal spine; (**b**) pieces of spine; (**c,d**) preparation to collagen extraction; (**e**) example of collagen extracted. (2) Analysis of whole spine: (**a**) spine; (**b**) steps to obtain powder of spine. (3) Powder of Opaque and Non-opaque bands: (**a**) section of the spine; (**b**) binocular to drilled the bands; (**c**) small drill; (**d**) powder obtained. (4) Weight in tin cups. (5) ThermoFisher Flash EA1112 elemental analyzer coupled to a Thermo Electron Delta Plus XP isotope ratio mass spectrometer (IRMS). (*Thunnus thynnus* picture permission granted by the author Andrea Paoli).

Dorsal fin spines were extracted and frozen prior to laboratory processing. For each tuna specimen, both the first dorsal spine (to determine δ^15^N and δ^13^C values) and the otoliths for age determination were extracted. After thawing, fin spines were cleaned of epidermal and dermal tissue and washed thoroughly with double-distilled water. The age of each specimen was determined by reading the growth increments in the otoliths, according to^39^. The laboratory analyses were performed following the work scheme showed in Fig. 6.

### Collagen extraction

Collagen was extracted from samples of Juveniles/Adults tuna through revised protocols used for fish bones^9,11^. In particular, for each specimen, the whole spine (having an average mass between 150 and 200 mg) was placed in a test-tube and 10 ml of 0.2 M HCl was added. The spine pieces were left for 24–48 h to demineralize at 5 °C. Demineralized residues were soaked overnight in 0.125 NaOH to remove lipids and other contaminants. These were then rinsed repeatedly in the filter funnel. Using a water bath, remaining residues were heated in centrifuge tubes for 10 h in a dilute HCl solution (pH~3) at 90–95 °C to solubilize collagen^9,40^. Finally, the remaining solution was concentrated on the ultra-filters by centrifugation at 2500 rpm. The supernatant of purified “collagen” was dried for 48 h, crushed and weighted (~0.5 mg) in tin cups.

### Whole spines and annuli sections

Whole spines of both YOY and Juveniles/Adults tuna were pulverized with mortar and pestle. Collagen and lipids were not removed from spines prior to isotope analysis. However, sub-sample of whole spine was subject to acidification (with 0.25 M HCl) to test the carbonate presence^16^. Additionally, growth layers of 7 sections of Juveniles/Adults tuna were also used for isotopic analysis. Opaque and non-opaque bands in otolith section were drilled with a small hand–held drill (Black&Deker RT650) under a binocular (Leica MZ6) and the obtained powder (~0.2 mg - 0.4 mg) was directly load in tin cups for isotopic analysis. The core of the section spine was also isotopically analyzed.

Collagen samples, whole spines (without collagen extraction), powder of opaque and non-opaque bands (untreated) were analysed with a ThermoFisher Flash EA 1112 elemental analyzer coupled to a Thermo Electron Delta Plus XP isotope ratio mass spectrometer (IRMS) at the Geochemistry Laboratory of the CNR Institute in Naples (Italy). Samples were run against blank cups and known urea standards (analytical grade urea of certificated isotopic composition) and IAEA international standard (IAEA N-1 and IAEA CH-7). Three capsules of urea were analysed at the beginning of each sequence. Moreover, one capsule of urea was analysed every six samples, in order to compensate for potential machine drift and as a quality control measure^41^. Experimental precision (based on the standard deviation of replicates of the internal standard) was <0.2‰ for δ^15^N and <0.1‰ for δ^13^C.

δ^13^C and δ^15^N values were obtained in parts per thousand (‰) relative to Vienna Pee Dee Belemnite (vPDB) and atmospheric N_2_ standards, respectively, according to the following formula:

$${{\rm{\delta }}}^{13}{\rm{C}}\,{\rm{or}}\,{{\rm{\delta }}}^{15}{\rm{N}}=[({{\rm{R}}}_{{\rm{sample}}}{/{\rm{R}}}_{{\rm{standard}}})-1]\times \,{10}^{3},\,{\rm{where}}\,{\rm{R}}={}^{13}{{\rm{C}}/}^{12}{\rm{C}}\,{\rm{or}}{}^{15}{{\rm{N}}/}^{14}{\rm{N}}$$

Results of isotopic values (δ^13^C and δ^15^N) are reported in Table 1. Where possible, replicates of analytical procedures and replicates of the same samples were performed. The standard deviation for each approach is also reported in Table 1.

When the percentage of total nitrogen (%N) was sufficient, C:N ratio was directly determined from isotopic analysis through the Elemental Analyzer using Acetanilide standard (C_8_H_9_ON, elemental composition: 71.09% carbon and 10.36% nitrogen).

### Statistical analysis

All statistical analyses were carried out by using R statistical environment (R Core Team, 2019). Statistical tests were used in order to verify if observed differences in terms of δ^15^N and δ^13^C between considered groups (collagen extracted *vs* non-extracted samples) were statistically significant. A preliminary check for normality and homogeneity of variances was carried out in order to verify the basic assumptions required by parametric t-test. If homogeneity of variances assumption was not met, the Welch t-test was used^42^, while, if normality assumption was not met, the non-parametric alternative t-test (i.e. the Wilcoxon tests) was adopted. When the number of groups was more than two, requiring then an ANOVA test, the same approach was adopted to check for normality and homogeneity of variances. If such assumptions were not met, the non-parametric Kruskal-Wallis ANOVA was used.

The relationship between δ^15^N values and FL was also investigated. In particular, the following exponential model was adopted:

$${\delta }^{15}N=a\ast (1-{e}^{(-FL/b)}).$$

The parameters estimate was performed by means of nonlinear (weighted) least-squares estimates by using the “nls2”^43^ package.

The FL value linked to each age was computed by means of the following age-length relationship^44^:

$$FL=373.08(1-{e}^{-0.07(t+1.76)}).$$

The nitrogen isotopic values at time t (δ_t_) or at the time t-1 (δ_t−1_) were also evaluated considering the isotopic mean of two consecutive bands (opaque and hyaline).
